# Development of In Situ Monitoring Sensor for Oil Spills in the Mediterranean Sea Using Portable Mass Spectrometry

**DOI:** 10.3390/molecules31091488

**Published:** 2026-04-29

**Authors:** Djordje Vujić, Milena Aleksić, Daria Ilić, Eleni Christoforou, Louis Hadjioannou, Boris Brkić

**Affiliations:** 1 BioSense Institute, University of Novi Sad, Dr Zorana Đinđića 1, 21000 Novi Sad, Serbia; milena.aleksic@biosense.rs (M.A.); daria.ilic@biosense.rs (D.I.); 2 Cyprus Marine and Maritime Institute, CMMI House, Vasileos Pavlou Square, Larnaca 6023, Cyprus; eleni.christoforou@cmmi.blue (E.C.); louis.hadjioannou@cmmi.blue (L.H.)

**Keywords:** MIMS, portable mass spectrometry, hydrocarbons, VOCs, Mediterranean sea

## Abstract

This study addresses the need for rapid, in situ detection of volatile organic compounds associated with oil contamination in aquatic environments. Membrane inlet mass spectrometry (MIMS) offers a direct, continuous monitoring approach without the need for chromatographic separation, making it suitable for real-time environmental analysis. In this work, a portable MIMS system was deployed at multiple pilot sites, including a fixed buoy platform, to evaluate its capability for detecting selected hydrocarbon target compounds under field conditions. Measurements were conducted over extended periods, and mass spectral data were continuously recorded and analyzed. Across all monitoring campaigns, no signals corresponding to the target analytes were observed above the established limits of detection. These findings demonstrate the robustness of the applied MIMS configuration for continuous environmental monitoring and confirm its suitability for detecting trace-level pollutants when present. The results also highlight the importance of field validation under realistic conditions, providing a basis for further optimization and broader application of MIMS in environmental surveillance.

## 1. Introduction

The Mediterranean basin is one of the most populous regions in the world, with one of the highest population densities globally. This trend is expected to continue, according to current projections [1,2]. Its geographical and cultural significance is best described by its own name, which derives from Latin and means “in the middle of the lands” of Europe, Asia, and Africa [2]. There is no doubt that this region has historically been, and will remain, of great importance to human societies. A concentrated population generates various socio-economic demands, which, in modern times, translate into intensive maritime traffic. Given that approximately 80% of global trade and 75% of Europe’s traded goods rely on maritime shipping [2,3], such extensive maritime activity is a major source of pollution and a threat to surrounding marine ecosystems. The largest share of pollution, approximately 45%, comes from operational oil discharges, followed by physical and chemical leaching, and about 8–10% comes from oil spill accidents. Operational discharges are continuous, and only regulations and a proper surveillance system can mitigate them [4]. However, oil spill accidents are not continuous, and depending on spill volume, they can inflict significant harm on surrounding areas. Studies show that 48% of all oil in the sea originates from fuels, while 29% is crude oil [5]. The 2025 report by the European Environment Agency and the European Maritime Safety Agency identified the North Sea and the Mediterranean Sea as regions at high risk of oil spills and illegal operational discharges due to intense maritime traffic [3]. Within the Mediterranean basin, major port cities are the most affected, especially those in the eastern part, as the region serves as a transit hub between Middle Eastern and Russian oil producers and Western European consumers [6]. Therefore, Cyprus, which was selected as the pilot site for this study, is a representative and relevant choice for oil spill monitoring.

Since the first serious tanker accident in 1967 in the English Channel, when almost 120,000 tons of oil were spilled into the marine environment, public awareness of this cause of pollution has increased [7]. Hence, significant effort is directed toward the prevention and early detection of accidents. Current information on accidental oil spill events is available through dedicated maritime databases and statistical resources, such as EMSA platforms and ITOPF tanker spill statistics [8,9]. All these resources facilitate locating accidents, but determining the sources remains a challenge. Additionally, these databases are not standardized globally [6]. Therefore, new monitoring technologies that can provide fast, reliable information about oil spill events are highly desirable.

Currently, both operational and accidental oil spills are monitored mainly using SAR (spaceborne synthetic aperture radar) and optical sensors, such as multispectral and hyperspectral imaging, as well as active sensors such as ultraviolet and infrared lasers [4,5,10]. These are mainly suitable for spatial detection and large-scale monitoring. However, they have difficulty distinguishing real oil from look-alikes, such as natural surface films of biological origin [4]. There have been efforts to introduce sensors that could provide more precise and accurate oil detection, such as UV-induced fluorescence sensors, which could distinguish different oil types [10]. However, without direct determination of composition, the accuracy of current approaches remains questionable.

Oil spill events release large quantities of volatile hydrocarbons such as n-alkanes, branched alkanes, cyclic alkanes, alkyl-benzenes, and polycyclic aromatic hydrocarbons (PAHs) [11]. Volatile organic compounds (VOCs) rapidly evaporate during the first hours to days following a spill accident and represent the dominant early-stage environmental exposure pathway [12]. During one model evaluation, ref. [13] demonstrated that within the first 30 h after an oil spill accident, evaporation is dominated by hydrocarbons containing fewer than 13 carbon atoms, followed by those with a higher number of carbon atoms. This is consistent with reports showing that oil spill accidents predominantly release mono-aromatic compounds such as benzene, toluene, ethylbenzene, and xylenes, also known as BTEX, as well as light alkanes and cycloalkanes, naphthalene, and up to C4-alkylated naphthalenes [14,15,16]. These VOCs not only pose severe harm to marine organisms [17] but also evaporate into the atmosphere [2,12,13], potentially causing harmful effects on both the environment and human health. They are known to be carcinogenic, neurotoxic, and genotoxic, causing cardiovascular diseases (CVDs) and issues with the reproductive system, amongst others [18,19]. Therefore, BTEXs are an excellent choice as early indicators of oil spill accidents, providing insights into the source of the spill.

Membrane inlet mass spectrometry (MIMS) holds great potential for use in various areas of volatolomics [14]. This technique is unique due to its very simple sampling principle, as it relies on the process of pervaporation and selective adsorption, diffusion, and desorption of VOCs through a membrane [20,21]. This enables direct sampling of gaseous and liquid matrices without sample preparation [22]. Additionally, it allows for both real-time/online monitoring of selected ions or a range of ions in the *m*/*z* 1–300 range, as well as offline analysis. Compared to other analytical approaches commonly used in VOCs analysis—gas chromatography with mass spectrometry (GC-MS), proton-transfer-reaction mass spectrometry (PTR-MS), or selected-ion-flow-tube mass spectrometry (SIFT-MS)—MIMS provides similar sensitivity, which indicates suitability for the measurement of VOCs in water [14]. In oil analysis, various GC-MS approaches are used and remain the benchmark for comprehensive chemical characterization [11]. However, when analyzing target compounds as indicators of oil presence, MIMS provides similar analytical performance with significantly shorter analysis times, enables continuous monitoring and does not require laboratory conditions. This facilitates on-site decision-making, which has great potential for rapid response and mitigation during marine pollution events [14,23]. Moreover, MIMS has already been successfully employed for environmental VOC monitoring in air and water, online detection of hydrocarbons [24], crude oil in water [25], organic compounds in nuclear waste ponds [26] and dissolved gases in the ocean [27]. In the review by Chua et al. (2016), many applications of MIMS in underwater research are presented, confirming its robustness for long-term deployment and remote monitoring scenarios [28].

Therefore, this study aimed to develop and validate the potential field deployment of a MIMS sensor for continuous in situ detection of fuel-derived VOCs in marine waters. After laboratory calibration and determination of analytical performance, the system was evaluated at three locations in Cyprus (two marinas and an offshore buoy), including a controlled gasoline-spike validation. To the best of our knowledge, no studies have demonstrated the feasibility of mounting a membrane inlet mass spectrometer on a buoy for surface water analysis, which is one of the harshest environments in nature due to wind and wave exposure. This approach could enhance the monitoring of oil spills and related incidents. In addition, a low-cost 3D-printed interface facilitates rapid prototyping, straightforward customization, and cost reduction of components.

## 2. Results

### 2.1. Results from the Pilot Locations

**Figure 1:**
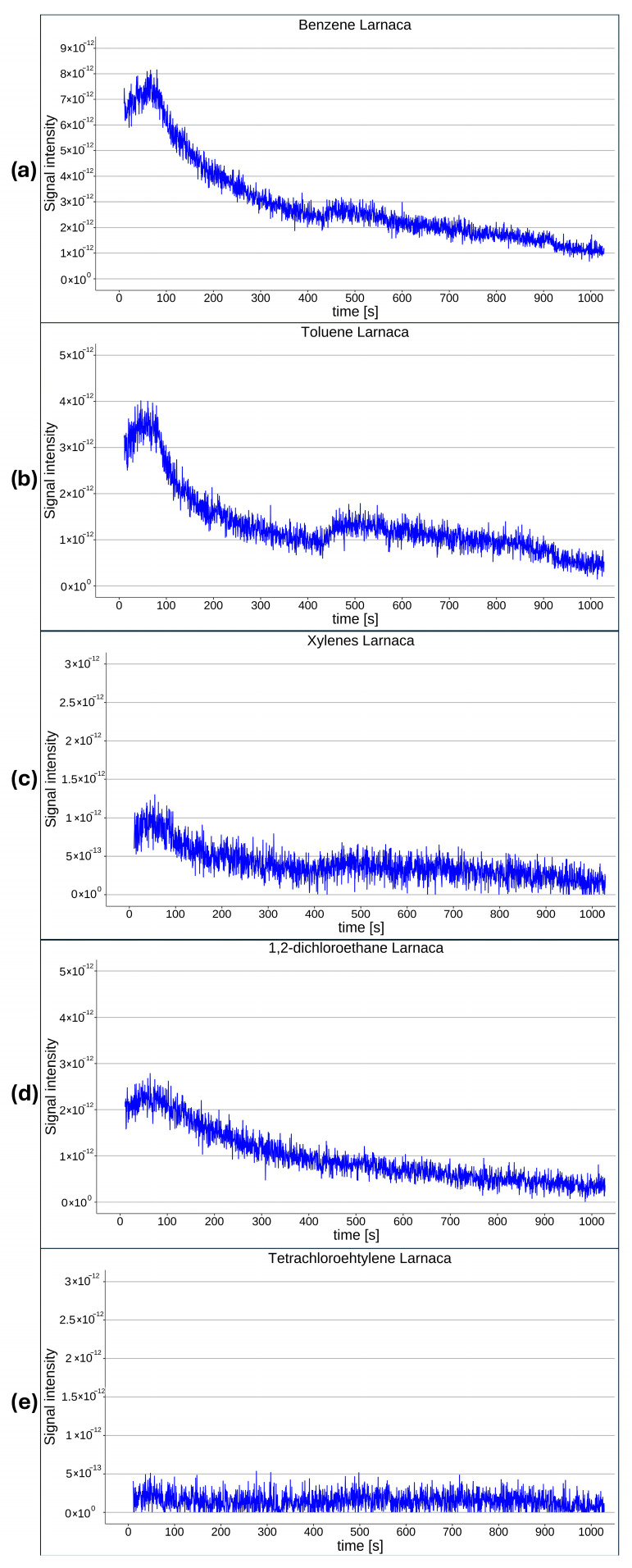
Test results from Larnaca Marina obtained using a portable MIMS system for benzene (**a**), toluene (**b**), xylenes (**c**), 1,2-dichloroethane (**d**), and tetrachloroethylene (**e**).

A key novelty of this study is that the tests were conducted in real marine environments rather than under controlled laboratory conditions. These deployments represent realistic, clean potential sampling locations and, therefore, constitute field validation of the proposed system. Across all three pilot locations, none of the investigated target compounds (BTX and selected chlorinated hydrocarbons) were detected at quantifiable levels. Signal intensities corresponding to characteristic *m*/*z* values for each compound remained below the established limits of detection (LODs) throughout the monitoring periods. Representative *m*/*z* intensities obtained at Larnaca Marina are shown in Figure 1. As illustrated, no distinct peaks attributable to the target analytes were observed during the monitored interval of 1000 s. These findings indicate the absence of detectable contamination by the investigated compounds under the prevailing environmental conditions and within the sensitivity limits of the current system configuration. The results, therefore, suggest that no recent fuel-related contamination occurred during the deployment period. Similar results were obtained in the St. Raphael marina and the offshore buoy; target compounds were either not present or below the LOD. The raw data used to generate the graphs presented in Figure 1 are provided in the Supplementary Materials as Datasets S1–S5.

### 2.2. In-Field Evaluation of Detection Performance

**Figure 2:**
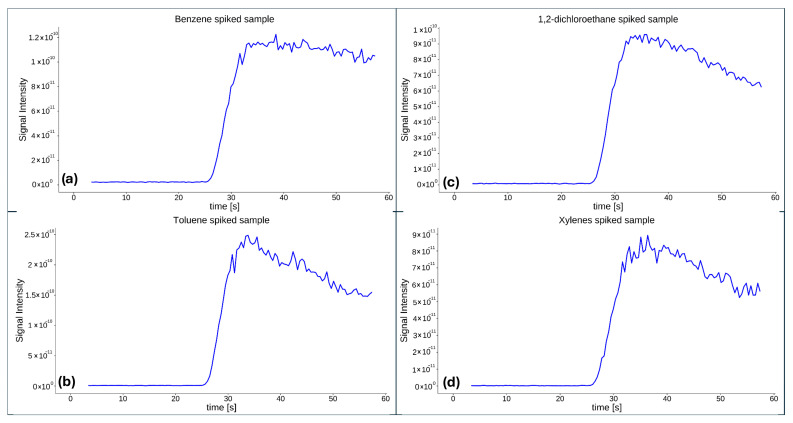
Signal for target compounds during the controlled experiment: (**a**) benzene, (**b**) toluene, (**c**) 1,2-dichloroethane, and (**d**) xylenes.

As no target compounds were detected at the three pilot sites, a controlled field test was conducted to verify proper sensor functionality, membrane permeability, and other potential issues. Without prior cleaning or maintenance of the sensor, membrane, or sampling interface, the sensor was placed into a 30 L bucket containing marina seawater, with 10 mL of unleaded gasoline added. Under these controlled conditions, the sensor immediately detected the expected compounds. Clear spikes in signal intensities were observed for benzene, toluene, and xylenes. The signal observed at *m*/*z* values corresponding to 1,2-dichloroethane is most likely attributable to overlapping mass fragments originating from gasoline components, highlighting the limitations associated with the absence of chromatographic separation. These observations underscore the reduced compound-discrimination capability of direct MIMS analysis compared to GC-MS systems that incorporate chromatographic resolution. Nevertheless, the pronounced responses recorded for the remaining target compounds confirm proper instrument operation and demonstrate the system’s ability to detect VOCs at measurable concentrations. Figure 2 represents the signal profiles recorded during the controlled experiment. Raw data used to generate graphs presented in Figure 2 are provided in the Supplementary Materials as Datasets S6–S9.

## 3. Discussion

The finding that BTX and chlorinated solvents remained below the limit of detection in this study is consistent with previously reported background levels of dissolved mono-aromatic hydrocarbons in marine environments. Studies conducted in Mediterranean coastal waters and other marine systems indicate that, outside of acute pollution events [29,30], these compounds are typically present at very low concentrations, often at trace or sub-µg/L levels [31]. The present findings, therefore, align with established baseline conditions and suggest that no detectable contamination occurred during the monitoring period. The application of membrane inlet mass spectrometry (MIMS) demonstrated several advantages for environmental monitoring. Its rapid response time and high sensitivity to dissolved gases and volatile organic compounds enable near real-time detection, which is particularly important for early identification of contamination events involving BTX compounds [21]. Compared to conventional grab sampling followed by laboratory-based GC–MS analysis, MIMS significantly reduces the time between sampling and detection, thereby improving the potential for timely response and mitigation. Furthermore, the suitability of MIMS for continuous, in situ monitoring has been demonstrated in marine environments [32]. Importantly, it also reduces the sampling bias inherent to traditional sampling methods, in which volatile compounds can evaporate or be lost during handling and transport to the laboratory, thereby increasing the risk of underestimating BTX and other VOC concentrations. Its compact design and minimal sample preparation requirements facilitate deployment on automated platforms such as buoys, flow-through systems, and research vessels. In addition, the reduced influence of high-salinity matrices enhances its applicability in marine and groundwater systems [22]. These characteristics support the integration of MIMS into sensor networks and digital monitoring infrastructures, enabling high-frequency environmental surveillance.

Overall, the results confirm that MIMS is a robust and effective tool for monitoring trace-level contaminants in aquatic environments with strong potential for application in early-warning systems and environmental risk management.

## 4. Materials and Methods

### 4.1. Chemicals and Reagents

Benzene, toluene, xylenes (mixed isomers), 1,2-dichloroethane, and tetrachloroethylene were selected as target volatile organic compounds (VOCs). Analytical-grade standards (≥99.9% purity) were obtained from Sigma-Aldrich (Taufkirchen, Germany). Membranes were acquired from Technical Products Inc. of Georgia (Buford, GA, USA). Working standard solutions were prepared by serial dilution with ethanol. Stock solutions of the VOCs in ethanol were then used to spike water to the final concentrations required to create calibration curves. All solutions were prepared in sealed glass vials to minimize volatilization losses and were freshly prepared prior to analysis in the field, while the mass spectrometer was calibrated at the deployment site. HPLC-grade ethanol was used for dilution. BTX compounds are relevant to oil spill detection because they are part of the water-soluble aromatic fraction of petroleum and can serve as early indicators of recent fuel or oil contamination in water. Conversely, chlorinated hydrocarbons are typically associated with industrial solvent contamination. The details of the compounds are presented in Table 1 along with their molecular weights and target ions.

### 4.2. Instrumentation

**Figure 3:**
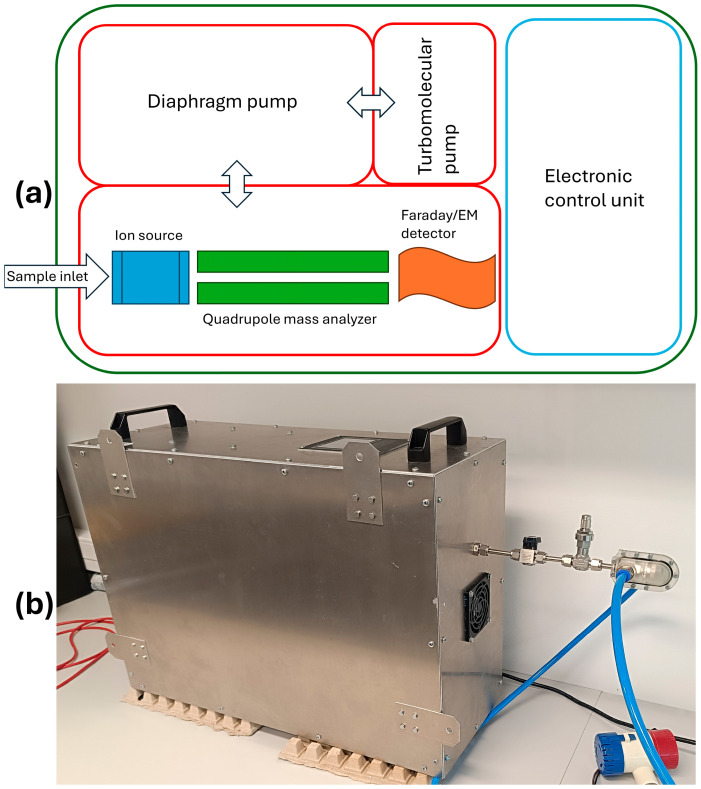
Schematic diagram of a portable oil sensor (**a**). Fully assembled prototype of the oil sensor system (**b**).

Mass spectrometric measurements were performed using a quadrupole mass spectrometer PrismaPro QMG 250 M3 (Pfeiffer Vacuum GmbH, Asslar, Germany). The instrument is equipped with an open ion source, a quadrupole mass analyzer and a Faraday/electron multiplier detector, allowing detection over a mass range of *m*/*z* 1–300. Ionization was achieved by electron ionization (EI) at 70 eV. The filament is made of tungsten coated with yttrium oxide. The filament current was set to 2000 µA. Mass spectra were acquired in selected ion monitoring (SIM) mode. The analyzer was operated under high-vacuum conditions maintained by a turbomolecular pump (HiPace 80) backed by a diaphragm pump (MVP 030-3DC), achieving a base pressure of approximately 4 × 10^−6^ Torr during operation, monitored by a digital cathode pressure gauge (MPT 200) all supplied by Pfeiffer Vacuum GmbH (Asslar, Germany). Support hardware included a submersible 12 V DC bilge pump, both during laboratory tests and pilot-scale trials in Cyprus. Data acquisition and instrument control were performed using PV MassSpec control software (V.23.06, Pfeiffer Vacuum GmbH, Asslar, Germany). Vacuum pumps were acquired from Pfeiffer Vacuum in Germany, and the vacuum chamber was custom-built by UltraHighVacuum in the UK. Operating conditions for the mass spectrometer are 5–50 °C. The mass spectrometer, vacuum system and support components were fitted inside an aluminum enclosure. The integrated system has overall dimensions of 25 × 45 × 60 cm (height × width × length) and weighs 27 kg. Figure 3 illustrates the system layout inside the enclosure and the fully assembled prototype.

### 4.3. Membrane Inlet and Sampling Interface

**Figure 4:**
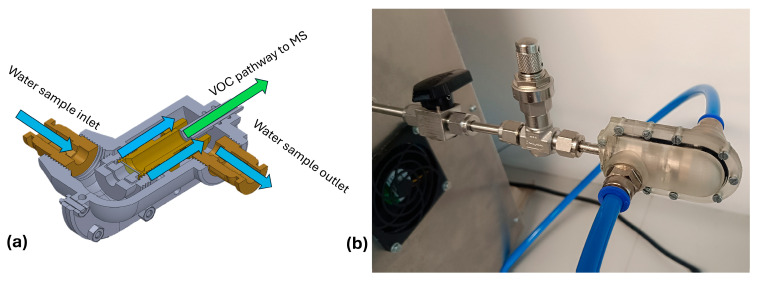
Sampling interface cross-section (**a**). Photograph of the sampling interface (**b**).

The mass spectrometer was interfaced with a membrane inlet sampling system designed for direct introduction of dissolved VOCs from aqueous samples. The membrane interface consisted of a polydimethylsiloxane (PDMS) membrane mounted within a custom-fabricated stainless-steel housing and a 3D-printed enclosure (see Figure 4). The 3D-printed enclosure was fabricated using high-temperature photopolymer resin (Form 3, Formlabs), enabling water circulation around the membrane housing. The membrane acted as a semi-permeable barrier, allowing selective diffusion of VOCs into the vacuum system while preventing water from entering the vacuum chamber. The effective membrane area was approximately 33 mm^2^, and the thickness was 127 µm. The membrane was supported on a porous disk to ensure mechanical stability under differential pressure conditions. The vacuum side of the membrane was directly connected to the ion source region of the quadrupole mass spectrometer. A continuous sample flow was maintained across the membrane surface using a submersible pump at a controlled flow rate of 2 L min^−1^. Initial lab tests showed the highest sensitivity at 2 L/min, at which point precise flow control via the voltage regulator was added to the system. The membrane interface was operated at a controlled temperature of 25 °C in lab tests to simulate the sea surface temperature at the test sites so that it maintained reproducible permeability and signal stability. All parts were constructed from chemically inert materials (e.g., PTFE hoses and stainless-steel housing) to reduce adsorption effects and cross-contamination. The sampling interface was designed to minimize dead volume and response time during measurements. Sampling at the marina sites was performed at a depth of approximately 20 cm below the water surface using a 4 m sampling hose. In contrast, sampling at the offshore buoy site was conducted at a greater depth due to restricted physical access from the buoy structure to the sea surface.

### 4.4. Calibration and Analytical Performance Evaluation

Calibration experiments were performed using aqueous standard solutions of benzene, toluene, xylene, 1,2-dichloroethane, and tetrachloroethylene prepared at multiple concentration levels within relevant working ranges. For each analyte, calibration curves were established by plotting the integrated ion signal intensities of selected characteristic *m*/*z* values against the corresponding nominal concentrations. Quantification was carried out by extracting compound-specific ions and directly determining the concentration from the calibration curve. Simple linear regression was used to obtain correlation coefficients. The limits of detection (LODs) and limits of quantification (LOQ) were determined using signal-to-noise (S/N) criteria (S/N = 3 for LOD and S/N = 10 for LOQ), calculated from ten consecutive measurements of the lowest calibration level. All data processing and regression analyses were performed using in-house software developed at the BioSense Institute.

Membrane inlet mass spectrometry (MIMS) has been reported to exhibit a linear response for VOCs across environmentally relevant concentration ranges, supporting its applicability for quantitative analysis.

**Figure 5:**
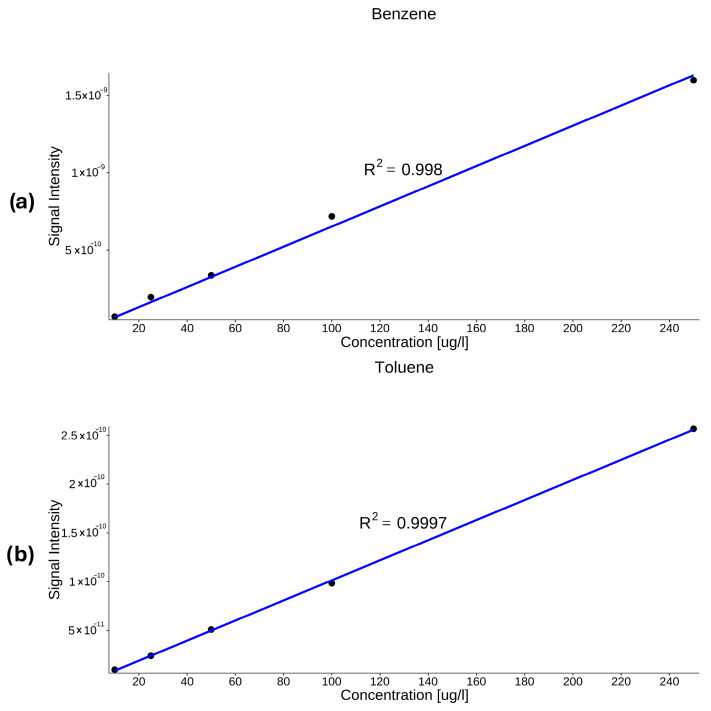
Calibration curves of benzene (**a**) and toluene (**b**).

**Figure 6:**
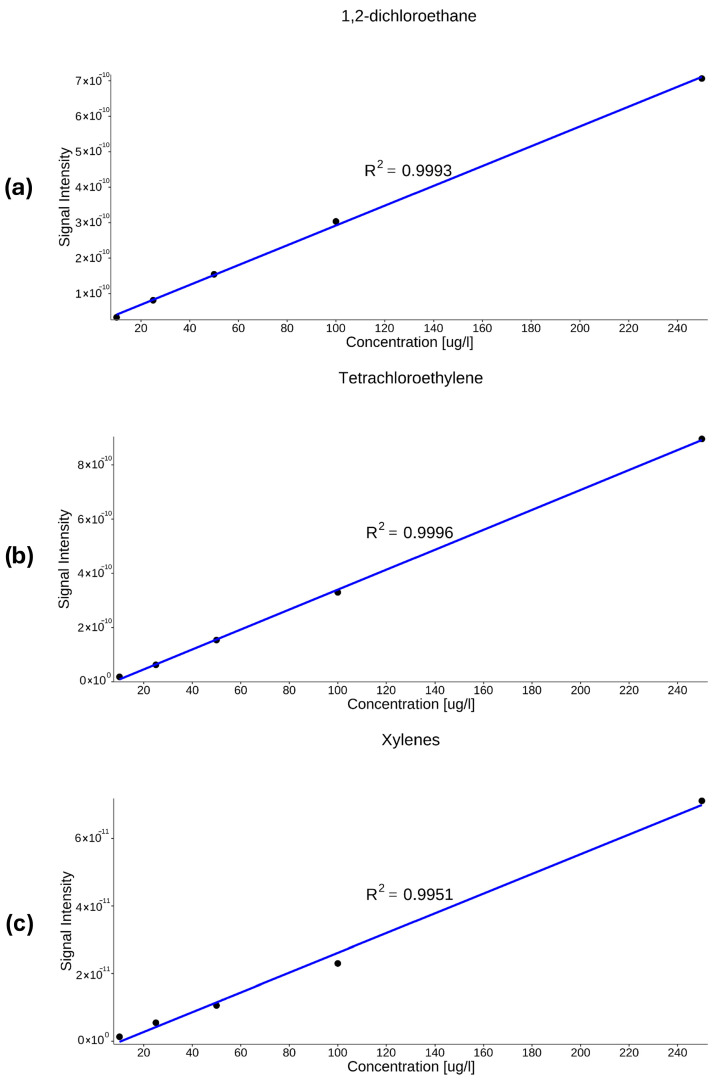
Calibration curves of 1,2-dichloroethane (**a**), tetrachloroethylene (**b**), and xylenes (**c**).

In previous studies, correlation coefficients (R^2^ > 0.99) have been described for aromatic hydrocarbons and related VOCs, while calibration curves were generated using the same methodology [33]. In the present study, strong linearity was observed for all investigated compounds, together with low limits of detection and quantification. The obtained analytical performance is consistent with previously reported MIMS-based determinations of dissolved VOCs. Figure 5 presents calibration curves for benzene and toluene, while Figure 6 shows calibration curves for xylenes, 1,2-dichloroethane and tetrachloroethylene. The raw data used to generate the calibration curves in Figure 5 and Figure 6 are provided in the Supplementary Materials as Datasets S10–S14.

Calibration curves exhibited linear behavior over the concentration range of 10–250 µg/L. Similar linear relationships were obtained for all the compounds (Table 2).

### 4.5. System Performance and Signal Stability

**Figure 7:**
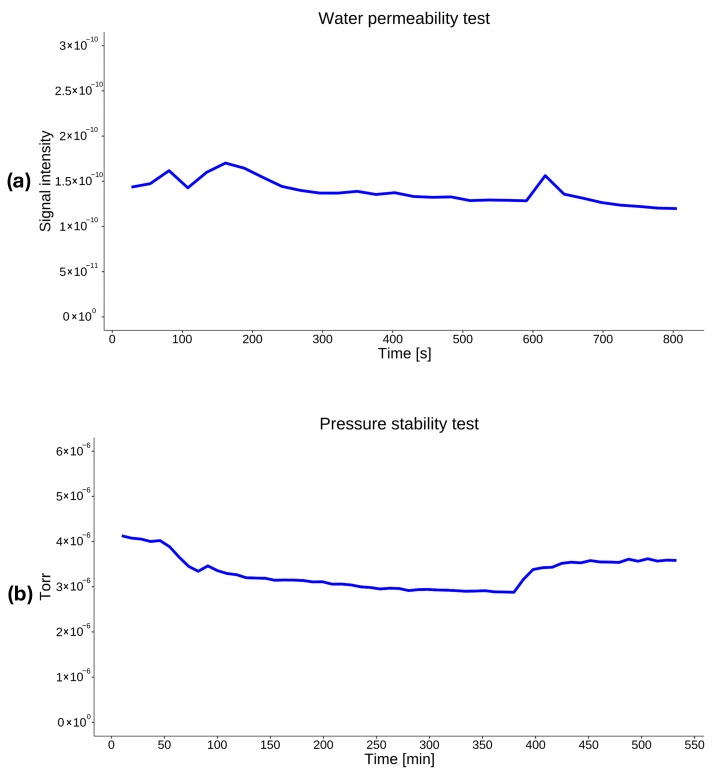
Water (**a**) and vacuum (**b**) stability tests.

The baseline stability of the system was evaluated in the laboratory under continuous operation using ultrapure water as the matrix. The water background signal was characterized by a dominant contribution at *m*/*z* 18 corresponding to the water vapor fragment and was monitored over a period of 20 min (see Figure 7a). Stable baseline conditions were observed, with signal fluctuations remaining within ±20% of the mean intensity. Water was chosen because it exhibited the highest drift and generated the most intense signal in the MIMS technique. This behavior is likely associated with its substantially greater polarity relative to the other analytes and the nonpolar characteristics of the membrane. No significant spontaneous signal spikes were detected during steady-state operation. Vacuum chamber pressure was monitored over a period of 10 h (see Figure 7b). The relative stability of these background ions confirms effective sealing of the membrane interface and stable vacuum conditions. Raw data used to generate graphs presented in Figure 7 are provided in the Supplementary Materials as Datasets S15 and S16.

**Figure 8:**
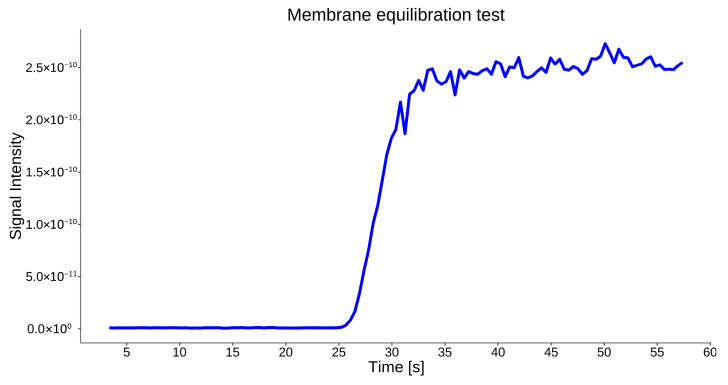
Membrane equilibration test for benzene.

Membrane equilibration behavior was assessed by monitoring signal response following initial immersion in aqueous samples. A stable signal plateau was typically achieved within approximately 1–5 min after immersion. Equilibration time depends on the analyte type and concentration, which directly influences gas diffusion across the PDMS membrane. After equilibration, the signal intensity remained stable under constant-flow conditions, as demonstrated for benzene in Figure 8. A memory effect of the membrane was also observed, as the system required between 1.5 and 6 min (depending on the type of analyte) to return to baseline after the sample was removed. Raw data used to generate Figure 8 is provided in the Supplementary Materials as Dataset S17.

### 4.6. Field Deployment–Pilot Experiments

**Figure 9:**
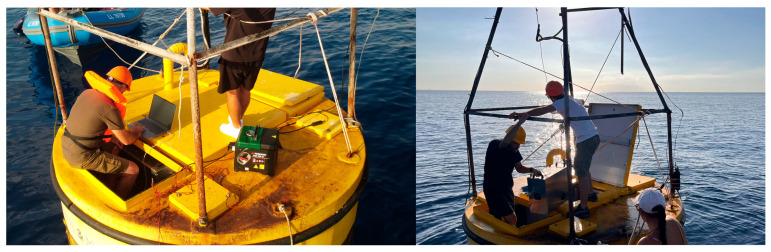
Installation of the oil sensor on the buoy during field deployment.

Field validation of the developed system was conducted at two marinas and one offshore buoy along the Mediterranean coast of Cyprus: Larnaca Marina, St. Raphael Marina, and a buoy ~2 km offshore from Limassol. Sites were chosen to cover different levels of oil pollution pressure, including areas influenced by routine vessel activity and areas with greater spill potential, ensuring that the seawater samples reflected conditions relevant to the sensor’s application. At each marina location, sample acquisition was performed over a period of 2 h under continuous-flow conditions. After the marina deployments, the sensor was installed on the buoy and operated continuously for two days, with seawater sample acquisition carried out for 2 h each day. This deployment was intended to assess the system’s extended operational stability and performance under open-sea conditions. Sampling was performed by continuously introducing surface seawater directly into the membrane inlet system, maintaining the same instrumental settings used during laboratory calibration. The sampling strategy aimed to capture spatial and temporal variability in VOC concentrations across semi-enclosed marina environments and open coastal waters. Environmental conditions during the deployments were typical for Cyprus in late September, characterized by relatively stable meteorological conditions and moderate sea temperatures (25–27 °C). The instrument was mounted on the buoy for two consecutive days. The sensor installation is shown in Figure 9. This robust system design enabled validation under realistic environmental conditions, including both enclosed marine waters and open-sea environments.

## 5. Conclusions

In this work, a compact membrane inlet mass spectrometry (MIMS) system was developed and validated for in situ monitoring of volatile organic compounds in marine environments. Laboratory calibration experiments demonstrated linear responses for BTX and selected chlorinated hydrocarbons, with high correlation coefficients and limits of detection in the low µg/L (ppb level) range, suitable for early detection of fuel and oil-related contamination. Field deployments at three locations in Cyprus (two marinas and one offshore buoy) confirmed the instrument’s stable operation under real marine environmental conditions, with continuous acquisition of background mass spectra and stable baselines. Although no BTX or chlorinated hydrocarbons were observed above the method detection limits during routine monitoring, a controlled gasoline-spike experiment at the St. Raphael Marina site produced clear responses for the target compounds, confirming the system’s capability to detect pollution events when they occur. Unlike gas chromatography–mass spectrometry (GC-MS), the presented MIMS configuration does not incorporate chromatographic separation, which may limit discrimination among compounds with overlapping mass fragments. However, the absence of a chromatographic column enables rapid response times and continuous in situ monitoring, representing a deliberate trade-off between selectivity and temporal resolution. Overall, the results indicate that the developed MIMS sensor is a promising tool for real-time surveillance of fuel-derived pollutants in coastal waters and marinas and provides a basis for future work on long-term deployments, enhanced selectivity strategies, and integration into broader marine monitoring networks and regulatory frameworks. To the authors’ knowledge, no studies have demonstrated the feasibility of deploying a membrane inlet mass spectrometer on a buoy platform for the identification of oil spills in surface water. However, this approach shows strong potential for advancing the monitoring of oil spills and related environmental incidents.

## Figures and Tables

**Table 1:** List of target compounds.

Compound	CAS Number	Molecular Weight (g/mol)	Target Ion (*m*/*z*)
Benzene	71-43-2	78.1	78, 77
Toluene	108-88-3	92.1	91, 92
Xylenes	1330-20-7	106.1	105, 106
1,2-dichloroethane	107-06-2	99.0	62, 64
Tetrachloroethylene	127-18-4	165.8	166, 164

**Table 2:** LOD, LOQ, linearity, and equations for target compounds.

Compound	LOD ^a^	LOQ ^a^	Equation	R^2^
Benzene	4.90	16.3	$Y=6.33\times 10^{-12}\times X+3.25\times 10^{-11}$	0.998
Toluene	7.50	24.8	$Y=1.03\times 10^{-12}\times X+\left(-1.72\times 10^{-12}\right)$	0.999
Xylenes	7.50	24.8	$Y=2.92\times 10^{-13}\times X+\left(-3.1\times 10^{-12}\right)$	0.995
Tetrachloroethylene	3.50	11.6	$Y=3.67\times 10^{-12}\times X+\left(-2.8\times 10^{-11}\right)$	0.999
1,2-dichloroethane	4.30	14.4	$Y=2.79\times 10^{-12}\times X+1.28\times 10^{-11}$	0.999

^a^ concentration in µg/L.

## Data Availability

The original contributions presented in this study are included in the article. Further inquiries can be directed to the corresponding author.

## Supplementary Materials

The following supporting information can be downloaded at https://www.mdpi.com/article/10.3390/molecules31091488/s1, Datasets S1–S5: raw instrument data (.dat files) used to generate graphs in Figure 1; Datasets S6–S9: raw instrument data (.dat files) used to generate graphs in Figure 2; Datasets S10 and S11: data used to generate calibration curves in Figure 5; Datasets S12–S14: data used to generate calibration curves in Figure 6; Datasets S15 and S16: raw data used to generate graphs in Figure 7; Dataset S17: raw data used to generate graph in Figure 8.

## Abbreviations

The following abbreviations are used in this manuscript:

BTX	Benzene, toluene, xylene
CVD	Cardiovascular disease
EI	Electron ionization
GC-MS	Gas chromatography–mass spectrometry
HPLC	High-performance liquid chromatography
LOD	Limit of detection
LOQ	Limit of quantification
MIMS	Membrane inlet mass spectrometry
PDMS	Polydimethylsiloxane
PTR-MS	Proton-transfer-reaction mass spectrometry
PTFE	Polytetrafluoroethylene
SIM	Selected ion monitoring
S/N	Signal-to-noise
VOC	Volatile organic compounds
